# Level of participation in physical therapy or an internet-based exercise training program: associations with outcomes for patients with knee osteoarthritis

**DOI:** 10.1186/s12891-018-2139-y

**Published:** 2018-07-19

**Authors:** Megan Pignato, Liubov Arbeeva, Todd A. Schwartz, Leigh F. Callahan, Jennifer Cooke, Yvonne M. Golightly, Adam P. Goode, Bryan C. Heiderscheit, Carla Hill, Kim M. Huffman, Herbert H. Severson, Kelli D. Allen

**Affiliations:** 1 University of North Dakota School of Medicine and Health Sciences, 1301 N Columbia Rd, Grand Forks, ND USA; 2 Thurston Arthritis Research Center, University of North Carolina at Chapel Hill, 3300 Thurston Bldg., CB#, Chapel Hill, NC 7280 USA; 3 Department of Medicine, University of North Carolina at Chapel Hill, 125 MacNider Hall CB#, Chapel Hill, NC 7005 USA; 4 Department of Biostatistics, Gillings School of Global Public Health, University of North Carolina at Chapel Hill, Chapel Hill, NC USA; 5 School of Nursing, University of North Carolina at Chapel Hill, Chapel Hill, NC USA; 6 Division of Physical Therapy, Department of Allied Health Services, University of North Carolina at Chapel Hill, Chapel Hill, NC USA; 7 Injury Prevention Research Center, University of North Carolina at Chapel Hill, Chapel Hill, NC USA; 8 Department of Epidemiology, University of North Carolina at Chapel Hill, Chapel Hill, NC USA; 9 Duke Clinical Research Institute, Durham, NC USA; 10 Division of Physical Therapy, Department of Orthopedic Surgery, Duke University Medical Center, Durham, USA; 11 Department of Orthopedics and Rehabilitation, University of Wisconsin-Madison, Madison, WI USA; 12 Division of Rheumatology, Department of Medicine, Duke University Medical Center, Durham, NC USA; 13 Physical Medicine and Rehabilitation Service, Durham VA Medical Center, Durham, NC USA; 14 Oregon Research Institute, Eugene, OR USA; 15 Center for Health Services Research in Primary Care, Durham VA Medical Center, Durham, NC USA

**Keywords:** Osteoarthritis, Exercise, Physical therapy

## Abstract

**Background:**

To examine whether number of physical therapy (PT) visits or amount of use of an internet-based exercise training (IBET) program is associated with differential improvement in outcomes for participants with knee osteoarthritis (OA).

**Methods:**

A secondary analysis was performed using data from participants in 2 arms of a randomized control trial for individuals with symptomatic knee OA: PT (*N* = 135) or IBET (*N* = 124). We examined associations of number of PT visits attended (up to 8) or number of days the IBET website was accessed during the initial 4-month study period with changes in Western Ontario and McMaster Universities Osteoarthritis Index (WOMAC) total, pain and function subscales, as well as a 2-min Step Test, at 4-month and 12-month follow-up.

**Results:**

Participants with more PT visits experienced greater improvement in WOMAC total score (estimate per additional visit = − 1.18, CI 95% = − 1.91, 0.46, *p* <  0.001) and function subscore (estimate = − 0.80, CI 95% = − 1.33, − 0.28, *p* <  0.001) across follow-up periods. For WOMAC pain subscale, the association with number of PT visits varied significantly between 4- and 12-month follow-up, with a stronger relationship at 4-months. There was a non-significant trend for more PT visits to be associated with greater improvement in 2-min Step Test. More frequent use of the IBET website was not associated with greater improvement for any outcome, at either time point.

**Conclusion:**

Increased number of PT visits was associated with improved outcomes, and some of this benefit persisted 8 months after PT ended. This provides guidance for PT clinical practice and policies.

**Trial registration:**

NCT02312713, posted 9/25/2015.

## Background

Osteoarthritis (OA) of the knee is a highly common condition, with a lifetime risk of occurrence of up to 45% [1]. Knee OA leaves many patients with pain, swelling and stiffness of the affected joint(s), all contributing to decreased function and quality of life [2, 3]. According to multiple guidelines, exercise is a core, first-line component for the management of OA [4]. This is based on evidence that multiple exercise-based interventions (structured exercise, general physical activity and physical therapy (PT)) can decrease pain while increasing physical function and quality of life [5–7].

Although there is strong evidence for the effectiveness of exercise-based interventions for knee OA, effects tend to be modest, and patients’ responses are variable [5]. One factor that may predict degree of improvement following an exercise-based intervention is participants’ level of participation in the intervention [8–10]. For example, one study among overweight adults with OA found greater exercise adherence (including attendance in group classes and completion of exercises at home) was associated with greater improvement in physical performance at short-term (6 month) and long-term (18 month) follow-up and a larger decrease in disability in the short-term [10]. Another study found that greater attendance during a 20-week aquatics exercise program for individuals with OA was associated with greater improvement in self-reported quality of well-being and depressive symptoms [8]. Although these studies indicate that degree of participation in structured, supervised exercise programs may influence the magnitude of impact, there is still a need for better understanding of whether and how this association may vary by format of exercise-based intervention, including those that are supervised (e.g. PT) and those that are self-directed.

Another important question is whether patient characteristics are associated with differential participation in exercise programs with different types of instruction. There has been relatively little study of patient characteristics that predict a greater level of participation in exercise-based interventions for patients with OA. However, prior studies suggest that the following factors are associated with level of adherence to exercise among individuals with OA: social support for exercise, self-efficacy for exercise, and better physcial and mental health [11–14]. Identifying patient characteristics that predict degree of participation in different types of exercise-based interventions for OA could help clinicians and researchers identify patients who may need additional support or determine which format of exercise-based intervention to recommend.

This study reports on secondary analyses from a 12-month randomized trial comparing PT to an internet-based exercise training program (IBET) for participants with symptomatic knee OA [15, 16]. In that study, we found that overall there were no statistically significant improvements in OA-related outcomes for either PT or IBET groups, with each compared to a usual care control group [15]. However, participants varied in both degree of participation in the interventions (e.g., number of PT sessions and amount use of the IBET program) and in the magnitude of improvement. Therefore, the first objective of these analyses was to examine whether level of participation in the interventions was associated with differential improvement in OA-related symptoms and function. This presents an importantopportunity to examine associations between treatment dose and outcomes in the context of two very different exercise-based interventions, including one that was closely supervised (PT) and one that was entirely self-directed (IBET). In addition, the study design allowed for examination of both short- (4-month) and long-term (12-month) outcomes. Our second objective was to examine whether participant characteristics were associated with degree of participation in the assigned intervention.

## Methods

### Participants and interventions

This study included participants from a study of Physical THerapy vs. INternet-Based Exercise Training for Patients with Knee Osteoarthritis (PATH-IN; NCT02312713); details of the study protocol and interventions have been previously reported [16]. PATH-IN participants were randomly assigned to one of three groups: PT, IBET, or wait list (WL) control. The PT intervention was modeled after standard care for patients with knee OA, with an emphasis on active interventions and a home exercise program; the intervention was delivered by physical therapists in multiple clinics. Based on a typical range of outpatient PT visits for knee OA, participants could receive up to 8 one-hour sessions during the initial 4 months of the study. Participants in the IBET group were given access to the website and encouraged to log on immediately after enrollment and to continue use of the program as often as possible throughout the study period. The IBET website provided an initial personalized exercise program, ongoing tailoring of exercise to facilitate appropriate progression and videos to demonstrate proper performance of stretching and strengthening exercises [17]. The website also sent reminders to participants via email after periods of not logging in.

Participants were identified through the University of North Carolina at Chapel Hill (UNC) and the Johnston County Osteoarthritis Project. All participants had a diagnosis of knee OA along with current joint symptoms. For the present study, only participants enrolled in either the PT (*N* = 140) or IBET (*N* = 142) groups were evaluated, albeit separately. Analyses were further restricted to participants who remained in the study at the 4-month assessment point, as these individuals had opportunity to fully participate in the PT intervention or had access to the IBET up to that time point. Five participants from the PT group were excluded because of developing health issues. In the IBET group, 12 participants withdrew and 6 participants were excluded due to health issues. Therefore, analyses included 135 participants from the PT group and 124 participants from the IBET group. This research is in compliance with the Helsinki Declaration and was approved by the Institutional Review Boards of UNC and Duke University Medical Center.

### Measures

#### Measures of participation in PT and IBET programs

For the PT group, we determined the number of visits attended by each participant, which was documented by the treating physical therapist in the study database. For the IBET group, we collected the number of days in which the participant logged onto the website within the first 4 months of the study. This was automatically tracked by the website. We also assessed self-reported physical activity, since home-based activity was a component of both programs. We administered the Physial Activity Scale for the Elderly (PASE), and for these analyses we used the Leisure Time Activity subscale, since it is of highest relevance (e.g., including performance of strengthening exercises) [18]. This subscale includes 6 items, with higher scores indicating more activity.

#### Measures of intervention effectiveness

##### Western Ontario and McMasters universities osteoarthritis index (WOMAC)

The WOMAC scale consists of 24 items covering three areas: pain, stiffness, and function. Each response category is answered using an ordinal scale: 0 (no symptoms) – 4 (extreme symptoms), therefore higher scores indicate worse symptoms [19]. Multiple studies have confirmed the validity and responsiveness of this measure [19]. The WOMAC total score, as well as pain and function subscales, were each included as outcome measures in this study.

##### Objective physical function – 2-minute step test

The 2-min Step Test requires the participant to step in place by bringing their knees to a height that is halfway between the iliac crest and patella. This test assesses lower extremity strength and endurance, based on the number of steps the participant is able to complete during a 2-min time period. This test has been validated as a measure of aerobic endurance [20].

#### Potential patient characteristics as predictors of PT and IBET use

We selected a group of demographic, clinical and psychosocial characteristics that have been associated with adherence to exercise or other behavioral therapies in prior studies [11–14].

##### Western Ontario and McMasters universities osteoarthritis index (WOMAC) and 2-minute step test

In analyses of factors predicting participation in either intervention (PT or IBET), the WOMAC pain and function subscales and 2-min Step Test (described above) were included as baseline characteristics.

##### Exercise self-efficacy

The Self-Efficacy for Exercise Scale (SEE) scale asks participants to rank their confidence, from 0 (not confident) to 10 (very confident), in their ability to complete exercise three times per week for 20 min each, in nine different contexts. The use of this scale has been validated by comparisons to expected associations with actual exercise [21].

##### Social support for exercise

The Social Support for Exercise Scale measures the degree to which participants perceive that they obtain support from friends or family to exercise. There are 10 items participants rank, on a scale of 1 (none) to 5 (very often), on the amount of support they receive from family or friends (separately) during the past 3 months. This scale was found to have good reliability and construct validity [22]. In addition, it has been shown to correlate well with exercise habits of participants [22].

##### Comfort with internet use

Participants’ comfort with internet use was measured via a survey. They were asked to rate how comfortable they were using the internet on a scale of 1 (not at all) to 5 (very).

##### Participant characteristics

The following characteristics were collected at baseline: age, gender (male/female), race (Non-white or White), highest level of education (any education less than a bachelor’s degree versus a bachelor’s degree or post-graduate work), body mass index (BMI), self-rated health (excellent, very good or good vs. fair or poor), and work status (employed versus not-working).

##### Data analysis

Descriptive statistics were computed overall and by study arm. Means and standard deviations were calculated for continuous variables and frequencies and percentages for categorical variables. The remainder of the analyses were performed for study arms separately. Repeated measures models were fitted as linear mixed effects models with change from baseline for each outcome as the dependent variables across both follow-up visits, accounting for the within-participant correlation. For each outcome variable, the model included baseline level of the respective outcome variable, time in months, the level of participation variable (number of PT visits or number of days on IBET website), and the interaction between time and the participation variable as the explanatory variables. A main effects model was also fitted (with the interaction term dropped from the model as specified above); the results for the participation variable term in these models was interpreted as reflecting the homogeneous association between the applicable participation variable and the respective outcome across both follow-up time points. To assist with interpretation of significant interactions, line graphs were created by plotting predicted changes from the regression equations. Graphs depicted the association of the participation variable with the outcome at 4- and 12-months. For descriptive purposes, we also performed a categorical analysis for the PT group. These participants were grouped into those who completed less than 2 visits, two to five visits, and more than six visits. This grouping was based on the distribution of the data, to achieve reasonable cell sizes; additional sensitivity analyses with different groupings of PT visits produced very similar results. Model-predicted mean estimates were calculated, along with 95% confidence intervals, at the 4- and 12-month follow-up visits separately for these 3 groupings.

Additionally, we conducted exploratory analyses with the participation variables and PASE Leisure Activity subscale as response variables and participant baseline characteristics treated as explanatory variables. These analyses included the WL group in addition to the PT and IBET groups, since the PASE was available for all 3 groups. First, bivariate analyses were conducted, using negative binominal regression models for the participation variables, and linear regression models for the PASE Leisure Activity subscale. For the latter, a logarithm transformation was used to improve the distribution of the residuals with respect to the normality assumption. Then, we included all explanatory variables achieving a significance level <  0.15 in the bivariate analyses in a corresponding multivariable regression model.

## Results

### Participant characteristics

The mean age of participants was 64.9 years (standard deviation (SD) = 10.9) and 70.7% were female. Fewer than half of the participants (40.9%) were employed at the start of the study. Of the participants within the IBET group one-third (36.3%) were of non-white race, whereas only one-fifth (21.1%) of participants within the PT group identified with this classification. Additional participant characteristics are displayed in Table 1.

**Table 1:** Participant Characteristics at Baseline^a^

Characteristic	All Participants (*N* = 259)	Internet-Based Exercise Training Group (*N* = 124)	Physical Therapy Group (*N* = 135)
Age, years	64.9 (10.9)	64.3 (11.5)	65.5 (10.4)
Female, N (%)	183 (70.7%)	85 (68.5%)	98 (72.6%)
Non-White Race, N (%)	73 (28.4%)	45 (36.3%)	28 (21.1%)
Married or Living with Partner, N (%)	158 (61%)	83 (66.9%)	75 (55.6%)
Bachelors Degree or Post-Graduate Education, N (%)	152 (58.7%)	68 (54.8%)	84 (62.2%)
Employed, N (%)	106 (40.9%)	47 (37.9%)	59 (43.7%)
Fair or Poor Health, N (%)	34 (13.1%)	21 (16.9%)	13 (9.6%)
Body Mass Index, kg/m^2^	31.7 (8.3)	31.8 (7.8)	31.7 (8.7)
WOMAC Total	31.5 (17.5)	31.6 (17.9)	31.4 (17.3)
WOMAC Pain Subscale	6.1 (3.7)	6.2 (3.9)	6 (3.4)
WOMAC Function Subscale	22.1 (12.7)	22 (12.9)	22.2 (12.6)
2-Minute Step Test	52.2 (30.4)	51.9 (29.9)	52.5 (30.9)
Social Support for Exercise Scale	51.8 (18.2)	52.1 (19.4)	51.6 (17.2)
Self-Efficacy for Exercise Scale	57.1 (20.1)	57.3 (19.7)	57 (20.5)

Missing Data: Race-2, WOMAC Total − 2, WOMAC Function – 2, PASE-8, PASE household-5, PASE leisure-5, Up and Go −3

*WOMAC* Western Ontario and McMaster Universities Osteoarthritis Index, *PASE* Physical Activity Scale for the Elderly

^a^Values are Mean (SD) unless otherwise specified

### PT group: Associations of number of visits with outcomes

**Fig. 1:**
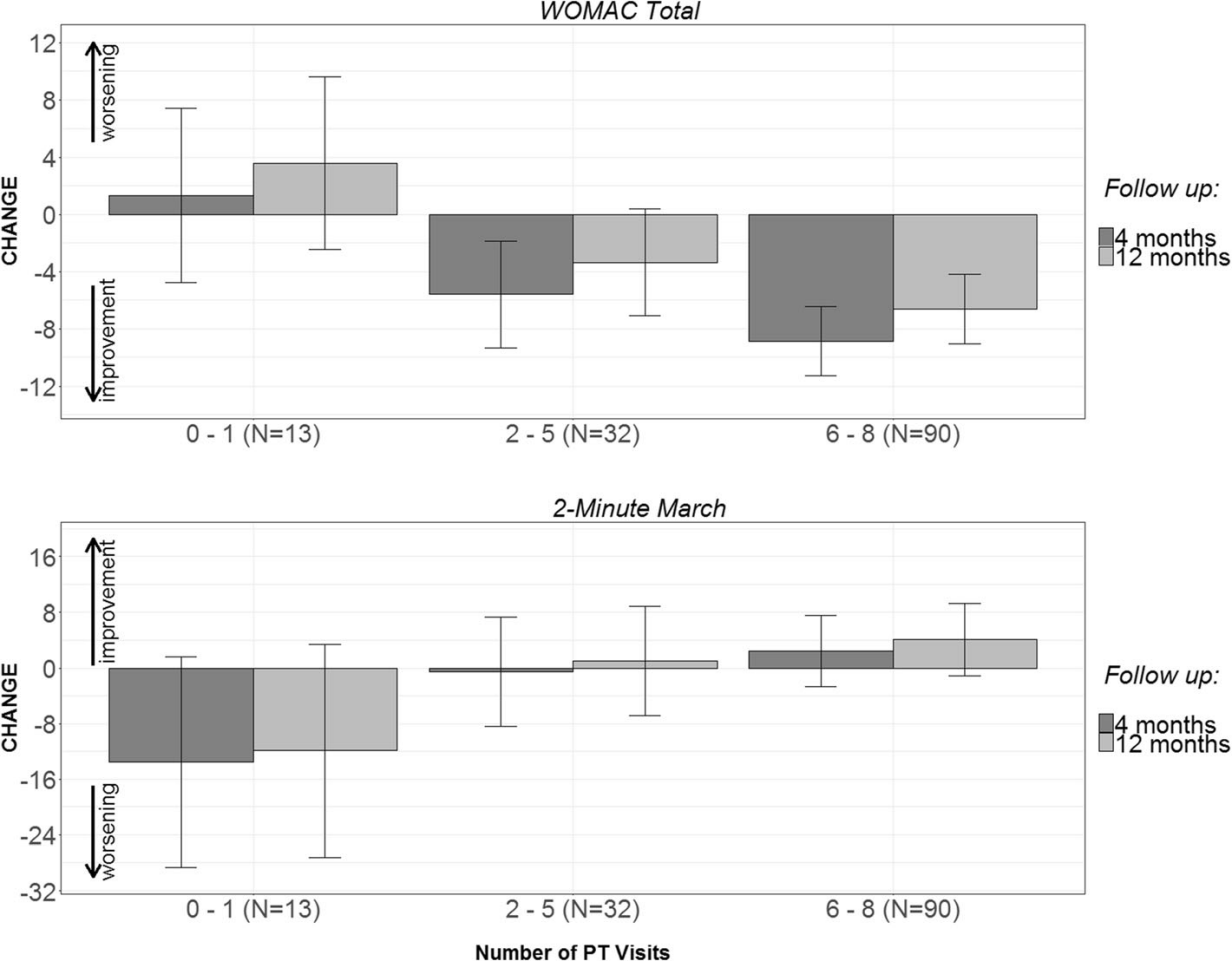
Model-Predicted Mean Changes in Outcome by Number of PT Visits Attended

**Fig. 2:**
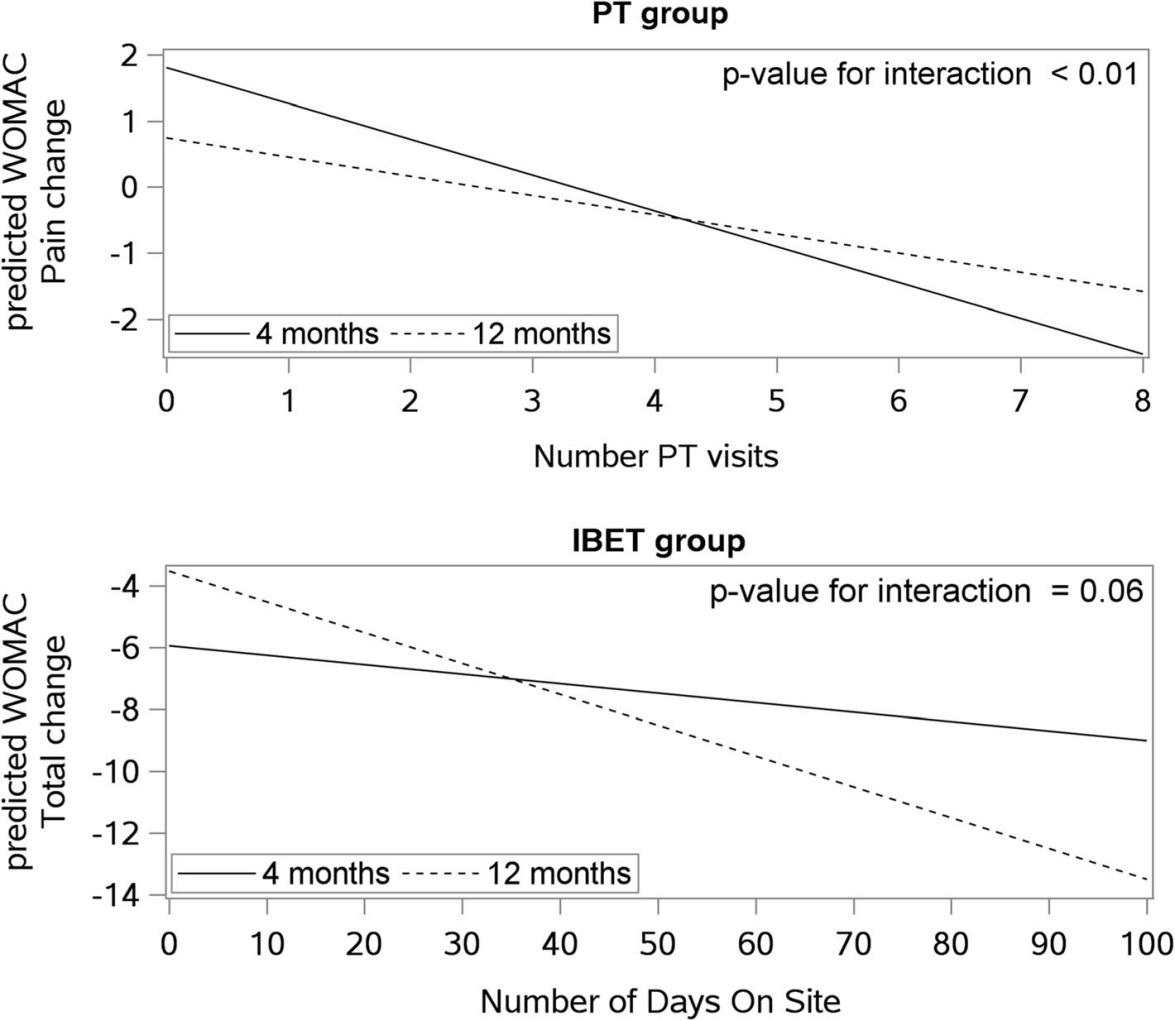
Associations between Use of Physical Therapy (PT) or Internet-Based Exercise Training (IBET) and change in Western Ontario and McMasters Universities Osteoarthritis Index Pain and Total Scores at 4- and 12-month Follow-up

The mean number of PT visits was 5.7 (SD = 2.5), with a median of 7 visits. In the repeated measures models for WOMAC total and function scores, the interactions between the number of PT visits and time were not significant (*p* > 0.05), indicating that the association between PT visits and the respective outcome did not vary significantly between the two follow-up time points. For both WOMAC total and function scores, a greater number of PT visits was associated with greater improvement (decreased score) at follow-up (Table 2). Figure 1 shows the mean WOMAC total scores based on number of PT visits attended. Participants who attended 0–1 PT visits had increases in WOMAC total score, while those who attended 2–5 or 6–8 PT visits had decreases in WOMAC total score at both time points, with those in the 6–8 visit group experiencing the greatest improvement. For the 2-min Step Test, the interaction between the number of PT visits and time was not significant. There was a marginally significant (*p* = 0.05) association between the number of PT visits and the increase in 2-min Step Test score at follow-up, regardless of time point. A similar pattern was observed for 2-min Step Test score as compared to WOMAC total score: participants who attended 6–8 PT visits demonstrated the greatest improvements at the 4- and 12-month follow-ups (Fig. 1). In regard to WOMAC pain, the interaction between the number of PT visits and follow-up time was significant (*p* <  0.05). As shown in Fig. 2, the slope was steeper at 4 months compared to 12 months, indicating a stronger association between the number of PT visits and change in WOMAC pain score at 4 months when compared to 12 months.

**Table 2:** Results from Repeated Measures Models for Each Outcome with of Number of PT Visits

		Model without Interaction		Model with Interaction	
		Estimate (95% CI)	*p*-value	Estimate (95% CI)	*p*-value
WOMAC Total Score	# PT visits	−1.18 (−1.91, − 0.46)	0.0015	− 1.97 (− 3.14, − 0.80)	< 0.01
	# PT visits * time	–	–	0.10 (− 0.02, 0.21)	0.10
WOMAC Function Score	# PT visits	− 0.80 (− 1.33, − 0.28)	0.003	− 1.29 (− 2.17, − 0.42)	< 0.01
	# PT visits * time	–	–	0.06 (− 0.03, 0.15)	0.17
WOMAC Pain Score	# PT visits	− 0.29 (− 0.46. -0.11)	0.001	− 0.54 (− 0.80, − 0.29)	< 0.01
	# PT visits * time	–	–	0.03 (0.01, 0.05)	< 0.01
2-Minute Step Test	# PT visits	1.57 (− 0.01, 3.15)	0.05	0.99 (−1.92, 3.90)	0.50
	# PT visits * time	–	–	0.08 (− 0.24, 0.40)	0.64

*N* = 135 for WOMAC Pain and 2-min; *N* = 134 for WOMAC Total and Function

### IBET group: Associations of numbers of days on website and outcomes

During the first 4 months, the mean number of days participants logged onto the IBET website was 20.7 (SD = 24.6), median = 9.5. During the full 12-month period, the mean number of days participants logged on was 40.5 (SD = 59.8), median = 10.5. In the repeated measures model of WOMAC total scores, there was a nearly statistically significant interaction between number of days on the website and follow-up visit (estimate per day on website = − 0.008, 95% CI = − 0.02 – 0.00, *p* = 0.06; Table 3). Plots of this interaction showed that the association between number of days on the IBET website and change in WOMAC total score was steeper at 12 months than at 4 months (Fig. 2); this indicates a stronger association at 12-month follow-up. For WOMAC function and pain scores, the interaction between number of days on the website and follow-up time was not significant. However, there were trends for the number of days on the website being associated with improvement in WOMAC function and pain score over time (*p* = 0.07 and 0.10, respectively) (Table 3). For the 2-min Step Test, the interaction between number of days on the website and time was not significant, nor was there an association between number of days on the website and change in the outcome over time in general.

**Table 3:** Results from Repeated Measures Models for Each Outcome with Number of Days on IBET Website

		Model without Interaction		Model with Interaction	
		Estimate (95% CI)	*p*-value	Estimate (95% CI)	*p*-value
WOMAC Total Score	# of Days on Website	−0.06 (− 0.14, 0.01)	0.10	− 0.03 (− 0.12, 0.05)	0.48
	# of Days on Website * time	–	–	− 0.01 (− 0.02, 0.00)	0.06
WOMAC Function Score	# of Days on Website	−0.05 (− 0.10, 0.005)	0.07	− 0.03 (− 0.10, 0.03)	0.32
	# of Days on Website * time	–	–	− 0.005 (− 0.01, 0.002)	0.17
WOMAC Pain Score	# of Days on Website	−0.02 (− 0.03, 0.003)	0.10	− 0.01 (− 0.03, 0.01)	0.44
	# of Days on Website * time	–	–	− 0.002 (− 0.004, 0.00)	0.10
2-Minute Step Test	# of Days on Website	0.09 (−0.06, 0.24)	0.24	0.05 (−0.12, 0.21)	0.56
	# of Days on Website * time	–	–	0.01 (−0.01, 0.03)	0.23

*N* = 124 in WOMAC Pain and 2-min; *N* = 123 in WOMAC Total and Function

### Associations of participant characteristics with level of participation in PT or IBET intervention

In bivariate analyses, the following characteristics were associated (*p* < 0.10) with greater number of PT visits attended: older age, non-white race, fair or poor self-rated health, and higher SEE; BMI and WOMAC pain were near the *p* < 0.10 threshold and were considered potentially important explanatory variables for number of PT visits (Table 4). In (Table 4). In multivariable analysis, only age was significantly associated (*p* < 0.05) with number of PT visits; specifically, older age was associated with greater number of PT visits attended (Table 4). When bivariate analyses were completed for the IBET intervention, there were no characteristics significantly associated with engagement to the IBET intervention. Since no variables met the criterion, a multivariable model was not completed.

**Table 4:** Results from Models for Associations of Number of PT Sessions Attended and Number of Days on the Website for IBET Intervention with Participant Characteristics

	PT Intervention						IBET Intervention		
	Bivariate Analysis			Multivariable Analysis			Bivariate Analysis^a^		
	Estimate	95% CI	*p*-value	Estimate	95% CI	*p*-value	Estimate	95% CI	*p*-value
Age (per 10 years)	0.12	0.05, 0.19	< 0.01	0.10	0.02–0.17	0.01	0.16	−0.07, 0.40	0.12
Female	0.02	−0.14, 0.18	0.82	–	–	–	0.11	−0.45, 0.67	0.69
Nonwhite race	0.28	0.09, 0.47	< 0.01	0.18	−0.04 – 0.41	0.11	0.27	−0.27, 0.81	0.32
Bachelors’ Degree or Post-Graduate Work	−0.04	−0.20, 0.11	0.57	–	–	–	−0.30	− 0.82, 0.22	0.26
Employed	0.03	−0.11, 0.18	0.66	–	–	–	0.04	−0.49, 0.57	0.88
Fair or Poor Self-Rated Health	0.38	0.09, 0.67	0.01	0.24	−0.08 – 0.56	0.13	0.12	−0.59, 0.82	0.75
BMI, kg/m^2^	−0.007	−0.02, 0.002	0.11	0.002	−0.01, 0.01	0.69	−0.02	−0.05, 0.02	0.30
WOMAC Pain Subscale	−0.0176	- 0.040, 0.005	0.12	0.004	−0.02, 0.03	0.77	−0.03	−0.10, 0.04	0.48
WOMAC Function Subscale	−0.003	−0.009, 0.002	0.26				−0.01	−0.03, 0.01	0.16
2-Minute Step	−0.001	− 0.003, 0.002	0.66	–	–	–	0.02	−0.004, 0.02	0.23
Social Support for Exercise	−0.003	−0.01, 0.002	0.24	–	–	–	0.003	−0.01, 0.02	0.72
Self-Efficacy for Exercise	0.004	0.00, 0.01	0.03	0.003	−0.001 – 0.01	0.11	0.01	−0.005, 0.02	0.23
Comfort with Internet Use	–	–	–	–	–	–	−0.03	−0.56, 0.49	0.90

*BMI* Body Mass Index

Missing data: race information was missing in 2 participants, Western Ontario and McMasters Universities Osteoarthritis Index (WOMAC) was missing in 2 participants

^a^Note: multivariable model is not applicable for the IBET intervention group based on the bivariate results

In bivariate analyses of the PT group, the following characteristics were associated (*p* <  0.15) with greater activity on the PASE Leisure Activity subscale: male gender, white race, greater education, lower BMI, better performance on the 2-min Step Test, and higher SEE (Table 5). In multivariable models among the PT group, greater education (estimate = 0.42, 95% CI = 0.07, 0.77, *p* = 0.02) and greater SEE (estimate = 0.01, 95% CI = 0.003, 0.018, *p* <  0.01) were associated with more activity on the PASE Leisure Activity subcale. In bivariate analyses for the IBET group, the following characteristics were associated (*p* <  0.15) with greater activity on the PASE Leisure Activity subscale: lower BMI, better scores on the WOMAC function subscale, better performance on the 2-min Step Test, higher social support for exercise, and higher SEE. In multivariable models among the IBET group, better performance on the 2-min Step Test (estimate = 0.007, 95%CI = 0.002, 0.013, *p* = 0.012), greater social support for exercise (estimate = 0.016, 95%CI = 0.008, 0.024, *p* < 0.001) and greater SEE (estimate = 0.010, 95% CI = 0.001, 0.018, *p* = 0.024) were associated with higher scores on the PASE Leisure Activity subscale. In bivariate analyses of the WL group, the following characteristics were significantly associated (*p* < 0.15) with greater activity on the PASE Leisure Activity subscale: male gender, currently working, lower BMI, better performance on the 2-min Step Test, and higher SEE; in multivariable models among the WL group, none of these variables were significantly associated (*p* < 0.05) with the PASE Leisure Activity subscale.

**Table 5:** Bivariate Associations of PASE Leisure Activity Subscale Scores with Participant Characteristics

	PT Intervention			IBET Intervention			Wait List Group		
	Estimate	95% CI	*p*-value	Estimate	95% CI	*p*-value	Estimate	95% CI	*p*-value
Age (per 10 years)	−0.06	−0.22, 0.10	0.48	−0.12	− 0.27, 0.04	0.14	− 0.04	−0.23, 0.16	0.70
Female	−0.52	−0.88, − 0.17	< 0.01 0.004	−0.23	− 0.60, 0.15	0.24	− 0.44	−0.97, 0.09	0.10
Nonwhite race	−0.42	−0.836, 0.002	0.05	−0.19	− 0.55, 0.17	0.30	− 0.21	−0.75, 0.34	0.45
Bachelors’ Degree or Post-Graduate Work	0.69	0.37. 1.02	< 0.001	0.27	−0.07, 0.62	0.12	0.16	−0.35, 0.66	0.54
Employed	0.06	−0.27, 0.40	0.71	0.15	−0.21, 0.50	0.41	−0.38	−0.84, 0.08	0.10
Fair or Poor Self-Rated Health	−0.36	−0.97, 0.25	0.25	−0.18	− 0.63, 0.28	0.45	− 0.31	−0.97, 0.35	0.35
BMI, kg/m^2^	−0.019	−0.039, 0.001	0.07	−0.03	− 0.046, − 0.004	0.02	−0.03	− 0.07, 0.01	0.10
WOMAC Pain Subscale	−0.03	−0.08, 0.02	0.27	−0.03	− 0.07, 0.02	0.23	− 0.01	−0.08, 0.05	0.75
WOMAC Function Subscale	−0.004	−0.017, 0.009	0.56	−0.02	− 0.030, − 0.004	0.01	−0.01	− 0.03, 0.01	0.29
2-Minute Step	0.009	0.004, 0.014	< 0.001	0.01	0.006, 0.017	< 0.001	0.01	−0.001, 0.016	0.08
Social Support for Exercise	0.001	−0.009, 0.010	0.82	0.02	0.10, 0.027	< 0.001	0.01	−0.005, 0.021	0.21
Self-Efficacy for Exercise	0.013	0.005, 0.021	< 0.01	0.014	0.005, 0.023	< 0.010	0.01	0.003, 0.025	0.01

## Discussion

This study assessed the association between degree of participation in two different exercise-based interventions, PT or IBET, and key OA outcomes. In addition, we evaluated associations between participant characteristics and participation in the interventions. There were three main findings of the study. First, results supported greater improvements in WOMAC total and function scores, as well as a positive trend for the 2-min Step Test, for participants who attended more PT visits; this association did not vary significantly between 4-month and 12-month outcomes. However, a greater number of PT visits was more strongly associated with 4-month than 12-month changes in WOMAC pain scores. Second, participants who logged onto the IBET website more frequently were not associated with improvements in study outcomes. Third, only one participant characteristic, older age, was found to be associated with number of PT visits attended, and none was found to be associated with number of days logging onto the IBET website.

### Association of Intervention Participation Level with effectiveness outcomes

In this study, participants who completed a greater number of PT sessions had overall better outcomes at follow-up. For one of our outcomes, WOMAC pain, greater number of PT visits had a stronger impact immediately following the completion of PT (4 months) than at a later time point. Importantly, however, for several outcomes (WOMAC total and function, 2-min Step Test), the association between number of PT visits and changes in outcomes did not differ significantly between the immediate post-treatment time point (4 months) and a later time point following an 8-month period of no study-delivered PT. The latter suggests that there may be a lasting, positive impact associated with receiving more PT visits on some outcomes. These results complement and expand on those of prior studies on exercise adherence and outcomes among patients with OA [8–10]. van Gool et al. also found that greater exercise adherence (comprised of class attendance and home exercise) had an impact on longer term (18-month) outcomes [10]. However, patients in that intervention continued to receive some support (either in-person or via telephone) throughout the 18-month period. Our study additionally suggests that even after a formal exercise-based intervention (specifically PT) has ended, the degree of earlier use of an intervention may predict longer term outcomes. Pisters et al. (2010) found that following discharge from a PT intervention, patients with greater adherence to prescribed home exercise experienced better outcomes [9]. It is possible that participants in our study who had more PT visits also adhered more to recommended home exercise and exercise-based therapies after completing PT, though this was not directly measured.

To date there are no established guidelines for the optimal number of outpatient PT visits for participants with knee OA [23–25]. Our results may provide information to help address this important question, which is highly relevant to both physical therapists and policy makers. In our analysis, participants who attended 0 or 1 PT visit experienced a worsening of their overall symptoms. In contrast, those with 2–5 and 6–8 visits showed clinically relevant improvement. Specifically, individuals with 2–5 visits had 18 and 11% improvement in WOMAC score at 4-month and 12-month follow-up, respectively. Individuals who attended 6–8 PT visits experienced even greater improvement: 28 and 21% at 4-month and 12-month follow-up, respectively. Prior research indicates that a 12% change in total WOMAC score represents a clinically relevant improvement in the context of this type of intervention [26]. These results suggest there may be added clinical benefit, even at 12-month follow-up, for providing 6–8 PT visits. It is likely that the optimal number of PT visits varies across patients, based on complexity, functional limitations and other factors. For some patients, it is possible that more than 8 visits would be beneficial.

The second main finding of this study was that although there was a trend for participants who logged into the website more frequently to have somewhat greater improvements in WOMAC total score, this association was small and not statistically significant. Therefore, associations of intervention participation level with outcomes differed between the supervised program (PT) and the self-directed program (IBET). One reason for the lack of association in the IBET group may be that use of the website overall was relatively low. Specifically, 28 of 142 participants never logged in, and the average (standard deviation) number of days participants logged in during the initial 4-month intervention period was 20.68 (24.62). Use of internet exercise interventions has varied across studies, with some showing higher rates than ours. Two studies that used similar web-based interventions for patients with arthritis had a greater number of logins, on average, as compared to our study [17, 27]. Although our study did not find greater use of the website to be associated with better outcomes, studies in other patient groups have found a positive association [28–30]. For example, a retrospective study completed by Hwang et al., found that participants who logged into a weight-loss website at least 4 times over a thirty-day period had significantly more weight loss than those whom had fewer logins [31]. Given the increasing use of internet-based and mobile health platforms to deliver health-related interventions, additional research is needed to identify best strategies for maximizing use of these types of interventions.

### Participant Characteristics Associated with Level of Intervention Participation

A third main finding of this study was that only one participant characteristic, older age, predicted more PT visits in adjusted analyses. It is possible that older participants had more flexible schedules that allowed them to attend the PT visits more easily, though we did control for working status. No participant characteristics were associated with amount of use of the IBET program. Therefore, this is another difference between the supervised program (PT) and the self-directed program (IBET). This lack of association for the IBET group could be due to the relatively low levels of engagement with the website, as described above. However, these results suggest that participants with a variety of demographic and personal characteristics may be similarly likely to engage with the website.

Greater baseline exercise self-efficacy was associated with more improvement in PASE Leisure Time Activity subscale scores, for both PT and IBET groups. This aligns with prior research regarding the important association of self-efficacy with adherence to exercise [11, 21, 32]. There were some differences in other predictors of change in PASE Leisure Time Activity scores across study groups. Although this may indicate that different patient characteristics are important for predicting improvement in activity for supervised (PT) vs. self-directed (IBET) programs, results should be considered in light of the exploratory nature of these analyses. In the WL group, no participant characteristics were associated with change in PASE Leisure Time Activity in multivariable analyses.

### Limitations

There are several limitations to this study. Although this study focused on important metrics of participation in exercise-based interventions, other components such as adherence to home exercise were not measured; this is an important area for future study. The interventions provided in this study were freely available to participants. Therefore, the degree of participation or factors associated with particiaption apply to situations when financial costs and co-payments are not barriers; in real-world settings, these are likely to be considerations for some patients and may affect participation levels. We purposefully chose to include in these analyses only participants who remained in the study until the 4-month follow-up, since they had opportunity to fully participate in the interventions during this time period. We acknowledge that this strategy omits some participants who never engaged with the interventions for a variety of reasons. We did not systematically ascertain reasons participants discontinued PT visits before the maxium allowed number, which may have included discomfort or lack of perceived benefit. Although this study was longitudinal in nature, there are still limitations to inferring that a greater number of visits caused greater improvement in outcome. Specifically, it is possible that patients who experienced more improvement in symptoms during the course of therapy were motivated to continue attending PT visits. We did not obtain de novo radiographs on participants, though all had a previous physician diagnosis and / or prior radiographic verification of knee OA. For our analyses of participant characteristics associated with treatment use, we were limited in the numbers of participant characteristics that could be included in multivariable models due to sample sizes. However, since few participant characteristics met our criterion in the bivariate analyses for inclusion in the multivariable model, this was not considered a major limitation. Finally, these were exploratory analyses with a large number of comparisons, and results should be interpreted with this in mind.

## Conclusion

In conclusion, this study found that increased number of PT visits resulted in better outcomes for participants, with the greatest improvements among those who attended 6–8 visits. These data can help to inform clinical practice and policies regarding insurance coverage for outpatient PT for management of knee OA. In addition, this information can be used by physical therapists to educate and encourage patients to persist in their PT process beyond a couple of initial visits. There were no significant improvements in outcomes for those who had greater use of the IBET program, but overall participation levels were low. Additional research is needed to identify best strategies for engaging patients with self-directed and mobile health interventions. Overall, participant characteristics were not strong predictors of degree of participation in either of these exercise-based interventions, potentially suggesting similar levels of acceptance for a variety of patients with knee OA.

## Abbreviations

IBET: Internet-Based Exercise Training
OA: Osteoarthritis
PASE: Phyiscal Activity Scale for the Elderly
PATH-IN: Physical THerapy vs. INternet-Based Exercise Training for Patients with Knee Osteoarthritis
PT: Physical Therapy
SD: Standard Deviation
SEE: Self-Efficacy for Exercise Scale
UNC: University of North Carolina at Chapel Hill
WL: Wait List
WOMAC: Western Ontario and McMasters Universities Osteoarthritis Index

## References

[1] Murphy L, Schwartz TA, Helmick CG, Renner JB, Tudor G, Koch G (2008). Lifetime risk of symptomatic knee osteoarthritis. Arthritis Rheum.

[2] Dominick KL, Ahern FM, Gold CH, Heller DA (2004). Health-related quality of life and health service utilization among older adults with osteoarthritis. Arthritis Care Res.

[3] Hootman JM, Helmick CG, Brady TJ (2012). A public health approach to addressing arthritis in older adults: the most common cause of disability. Am J Public Health.

[4] Nelson AE, Allen KD, Golightly YM, Goode AP, Jordan JM (2014). A systematic review of recommendations and guidelines for the management of osteoarthritis: the chronic osteoarthritis management initiative of the U.S. bone and joint initiative. Semin Arthritis Rheum.

[5] Fransen M, McConnell S, Harmer AR, Van der Esch M, Simic M, Bennell KL (2015). Exercise for osteoarthritis of the knee: a Cochrane systematic review. Br J Sports Med.

[6] Roddy E, Zhang W, Doherty M (2005). Aerobic walking or strengthening exercise for osteoarthritis of the knee? A systematic review. Ann Rheum Dis.

[7] Wang SY, Olson-Kellogg B, Shamliyan TA, Choi JY, Ramakrishnan R, Kane RL (2012). Physical therapy interventions for knee pain secondary to osteoarthritis: a systematic review. Ann Intern Med.

[8] Belza B, Topolski T, Kinne S, Patrick DL, Ramsey SD (2002). Does adherence make a difference? Results from a community-based aquatic exercise program. Nurs Res.

[9] Pisters MF, Veenhof C, Schellevis FG, Twisk JW, Dekker J, De Bakker DH (2010). Exercise adherence improving long-term patient outcome in patients with osteoarthritis of the hip and/or knee. Arthritis Care Res.

[10] van Gool CH, Penninx BW, Kempen GI, Rejeski WJ, Miller GD, van Eijk JT (2005). Effects of exercise adherence on physical function among overweight older adults with knee osteoarthritis. Arthritis Rheum.

[11] Marks R, Allegrante JP (2005). Chronic osteoarthritis and adherence to exercise: a review of the literature. J Aging Phys Act.

[12] Lee FI, Lee TD, So WK (2016). Effects of a tailor-made exercise program on exercise adherence and health outcomes in patients with knee osteoarthritis: a mixed-methods pilot study. Clin Interv Aging.

[13] Joseph RP, Dutton GR, Cherrington A, Fontaine K, Baskin M, Casazza K (2015). Feasibility, acceptability, and characteristics associated with adherence and completion of a culturally relevant internet-enhanced physical activity pilot intervention for overweight and obese young adult African American women enrolled in college. BMC Res Notes.

[14] Fernandopulle S, Perry M, Manlapaz D, Jayakaran P. Effect of land-based generic physical activity interventions on pain, physical function, and physical performance in hip and knee osteoarthritis: a systematic review and meta-analysis. Am J Phys Med Rehabil. 2017; 10.1097/PHM.0000000000000736. 10.1097/PHM.0000000000000736 28323761

[15] Allen KD, Arbeeva L, Callahan L, Golightly YM, Goode AP, Heiderscheit BC, et al. Physical therapy vs. internet-based exercise training for patients with knee osteoarthritis: results of a randomized controlled trial. Osteoarthr Cartil 2018;In Press. 10.1016/j.joca.2017.12.008 PMC6021028 29307722

[16] Williams QI, Gunn AH, Beaulieu JE, Benas BC, Buley B, Callahan LF (2015). Physical therapy vs. internet-based exercise training (PATH-IN) for patients with knee osteoarthritis: study protocol of a randomized controlled trial. BMC Musculoskelet Disord.

[17] Brooks MA, Beaulieu JE, Severson HH, Wille CM, Cooper D, Gau JM (2014). Web-based therapeutic exercise resource center as a treatment for knee osteoarthritis: a prospective cohort pilot study. BMC Musculoskelet Disord.

[18] Washburn RA, Ficker JL (1999). Physical activity scale for the elderly (PASE): the relationship with activity measured by a portable accelerometer. J Sports Med Phys Fitness.

[19] Bellamy N (2002). WOMAC: a 20-year experiential review of a patient-centered self-reported health status questionnaire. J Rheumatol.

[20] RIkli R, Jones J (1999). Development and validation of a functional fitness test for community-residing older adults. J Aging Phys Act.

[21] Resnick B, Spellbring AM (2000). Understanding what motivates older adults to exercise. J Gerontol Nurs.

[22] Sallis JF, Grossman RM, Pinski RB, Patterson TL, Nader PR (1987). The development of scales to measure social support for diet and exercise behaviors. Prev Med.

[23] Deyle GD, Allison SC, Matekel RL, Ryder MG, Stang JM, Gohdes DD (2005). Physical therapy treatment effectiveness for osteoarthritis of the knee: a randomized comparison of supervised clinical exercise and manual therapy procedures versus a home exercise program. Phys Ther.

[24] Jamtvedt G, Dahm KT, Christie A, Moe RH, Haavardsholm E, Holm I (2008). Physical therapy interventions for patients with osteoarthritis of the knee: an overview of systematic reviews. Phys Ther.

[25] McAlindon TE, Bannuru RR, Sullivan MC, Arden NK, Berenbaum F, Bierma-Zeinstra SM (2014). OARSI guidelines for the non-surgical management of knee osteoarthritis. Osteoarthritis Cartilage / OARS, Osteoarthritis Res Soc.

[26] Angst F, Aeschlimann A, Stucki G (2001). Smallest detectable and minimal clinically important differences of rehabilitation intervention with their implications for required sample sizes using WOMAC and SF-36 quality of life measurement instruments in patients with osteoarthritis of the lower extremities. Arthritis Rheum.

[27] Lorig KR, RItter PL, Laurent D, Plant K (2008). The internet-based arthritis self-Mangement program: a one-year randomized trial for patients with arthritis or fibromyalgia. Arthritis Care Res.

[28] Glasgow RE, Christiansen SM, Kurz D, King DK, Woolley T, Faber AJ (2011). Engagement in a diabetes self-management website: usage patterns and generalizability of program use. J Med Internet Res.

[29] Rotondi AJ, Anderson CM, Haas GL, Eack SM, Spring MB, Ganguli R (2010). Web-based psychoeducational intervention for persons with schizophrenia and their supporters: one-year outcomes. Psychiatr Serv.

[30] Patrick K, Calfas KJ, Norman GJ, Rosenberg D, Zabinski MF, Sallis JF (2011). Outcomes of a 12-month web-based intervention for overweight and obese men. Ann Behav Med.

[31] Hwang KO, Ning J, Trickey AW, Sciamanna CN (2013). Website usage and weight loss in a free commercial online weight loss program: retrospective cohort study. J Med Internet Res.

[32] Allegrante JP, Marks R (2003). Self-efficacy in management of osteoarthritis. Rheum Dis Clin N Am.

